# BEST PRACTICE CAREGIVING: DIFFERENCES AND GAPS AMONG DEMENTIA SUPPORT PROGRAMS FOR FAMILY & FRIEND CAREGIVERS

**DOI:** 10.1093/geroni/igz038.3471

**Published:** 2019-11-08

**Authors:** Julie H Rentsch, David Bass, Kathy Kelly, Katie Maslow, Alyssa Ciancibello, Leah Eskenazi, Rachel Schaffer

**Affiliations:** 1 Benjamin Rose Institute on Aging, Cleveland, Ohio, United States; 2 Family Caregiver Alliance, San Francisco, California, United States; 3 Gerontological Society of America, Washington, District of Columbia, United States

## Abstract

Family members and friends are the main providers of care for persons living with dementia. However, dementia caregivers are at greater risk than other caregivers of experiencing negative caregiving consequences. Despite the development of evidence-based programs to support dementia caregivers, few health or social service organizations offer any of these programs due, in part, to a lack of knowledge about their availability. Best Practice Caregiving is a newly launched website where professionals can get detailed information about these programs. Data collected to develop Best Practice Caregiving are analyzed for a sample of 42 evidence-based dementia caregiving programs to describe similarities and differences among programs including gaps in assistance available from these programs. Results show 64% of programs are delivered to caregivers only while the remaining are delivered to the caregiver and/or persons with dementia. Nearly half (43%) of the 42 programs are delivered in-person, 38% by phone, with 17% delivered all or in part online. Most programs are delivered by professionals (86%) followed by trained lay leaders (40%) and self-guided (12%). Most programs (95%) provide assistance with coping with illness/caregiving and the relationship of the dyad. Fewer than half of the programs assist caregivers with issues regarding finances (45%), end-of-life care (43%), and medical care (40%). Data from 233 delivery organizations show the most common challenge was getting caregivers to accept and complete the program (86%). Delivery sites reported more success with funding the program (mean=8.2 on a scale of 1-10) than with marketing and recruiting participants (mean=6.7).

